# Quality Assessment of Studies Published in Open Access and Subscription Journals: Results of a Systematic Evaluation

**DOI:** 10.1371/journal.pone.0154217

**Published:** 2016-05-11

**Authors:** Roberta Pastorino, Sonja Milovanovic, Jovana Stojanovic, Ljupcho Efremov, Rosarita Amore, Stefania Boccia

**Affiliations:** 1 Section of Hygiene—Institute of Public Health, Università Cattolica del Sacro Cuore, Rome, Italy; 2 Studies Coordinating Centre, Research Unit Hypertension and Cardiovascular Epidemiology, KU Leuven, Leuven, Belgium; 3 Section of Hygiene—Institute of Public Health, Università Cattolica del Sacro Cuore, Fondazione Policlinico Universitario “Agostino Gemelli”, Rome, Italy; NIH—National Institute of Environmental Health Sciences, UNITED STATES

## Abstract

**Introduction:**

Along with the proliferation of Open Access (OA) publishing, the interest for comparing the scientific quality of studies published in OA journals versus subscription journals has also increased. With our study we aimed to compare the methodological quality and the quality of reporting of primary epidemiological studies and systematic reviews and meta-analyses published in OA and non-OA journals.

**Methods:**

In order to identify the studies to appraise, we listed all OA and non-OA journals which published in 2013 at least one primary epidemiologic study (case-control or cohort study design), and at least one systematic review or meta-analysis in the field of oncology. For the appraisal, we picked up the first studies published in 2013 with case-control or cohort study design from OA journals (Group A; n = 12), and in the same time period from non-OA journals (Group B; n = 26); the first systematic reviews and meta-analyses published in 2013 from OA journals (Group C; n = 15), and in the same time period from non-OA journals (Group D; n = 32). We evaluated the methodological quality of studies by assessing the compliance of case-control and cohort studies to Newcastle and Ottawa Scale (NOS) scale, and the compliance of systematic reviews and meta-analyses to Assessment of Multiple Systematic Reviews (AMSTAR) scale. The quality of reporting was assessed considering the adherence of case-control and cohort studies to STrengthening the Reporting of OBservational studies in Epidemiology (STROBE) checklist, and the adherence of systematic reviews and meta-analyses to Preferred Reporting Items for Systematic reviews and Meta-Analysis (PRISMA) checklist.

**Results:**

Among case-control and cohort studies published in OA and non-OA journals, we did not observe significant differences in the median value of NOS score (Group A: 7 (IQR 7–8) versus Group B: 8 (7–9); p = 0.5) and in the adherence to STROBE checklist (Group A, 75% versus Group B, 80%; p = 0.1). The results did not change after adjustment for impact factor. The compliance with AMSTAR and adherence to PRISMA checklist were comparable between systematic reviews and meta-analyses published in OA and non-OA journals (Group C, 46.0% versus Group D, 55.0%; p = 0.06), (Group C, 72.0% versus Group D, 76.0%; p = 0.1), respectively).

**Conclusion:**

The epidemiological studies published in OA journals in the field of oncology approach the same methodological quality and quality of reporting as studies published in non-OA journals.

## Introduction

Over the last 25 years, scientific journal publishing has undergone a veritable transformation, enabled by the technical potentials offered by the Web. First of all, electronic publishing has become the prevalent distribution channel for scholarly journals. Additionally, the Open Access (OA) system was launched allowing researchers to access scientific publications without any restrictions posed by subscriptions. A core concept of OA journal publishing is a transition from subscription fees to alternative ways of funding publication, and since the early 1990s OA journal publishing has been growing at a far faster rate than traditional subscription journal publishing [1].

In 2000, two new publishers, the Public Library of Science (PLoS) and BioMed Central (BMC), launched the use of article processing charges (APC) as the central means of financing professional publishing OA journals. While the traditional model relies on restricting access to published research in order to recoup the costs of the publication process, the OA publishing model treats publication as the last phase of the research process and the APC is levied at the beginning of the process. To date the number of OA journals is 10,249 [2].

Several authors, however, have debated whether the proliferation of OA publishing would damage the peer review system and put the quality of scientific journal publishing at risk [3–10]. There is the perception that if journals collect fees from authors rather than subscribers, those journals will be inclined to accept substandard articles since their income is linearly dependent on the number of published studies.

A commentary published on Science in 2013 documented little or no scrutiny of many OA journals [10]. The author, under a false identity, submitted 304 versions of a flawed manuscript to OA journals during a 10-months period. More than 50% of those journals accepted the manuscript, and in 60% of the cases the decision was made without any formal peer review process.

The scientific quality of the scholarly journals is a difficult concept to quantify. There are some previous studies that attempted to determine the quality of publications from OA journals compared to traditional subscription journals [11–17]. The proxy used for the quality assessment was the number of citations, and results show that the average citation rates, after controlling for the number of articles, discipline, age of the journal and the location of the publisher are comparable between OA and subscription journals [12,16,17]. Our study aims to compare the methodological quality and the quality of reporting of scientific publications from OA versus non open access (non-OA) journals, by using validated scales. To this end we included primary epidemiological studies and systematic reviews and meta-analyses published in the field of oncology in 2013.

## Methods

### Journals and study inclusion criteria

The list of medical journals in the field of oncology was acquired from Thomson Reuters Intellectual Property and Science using the proper code field, by accessing http://science.thomsonreuters.com/cgi-bin/jrnlst/jlresults.cgi?PC=D&SC=DM in January 2014.

We classified journals as OA if there was a compulsory fee to pay for publishing (without making a distinction between for-profit and not-for-profit OA journals), and non-OA journals if there was a voluntary fee to pay for a free download of the published full text.

**Fig 1:**
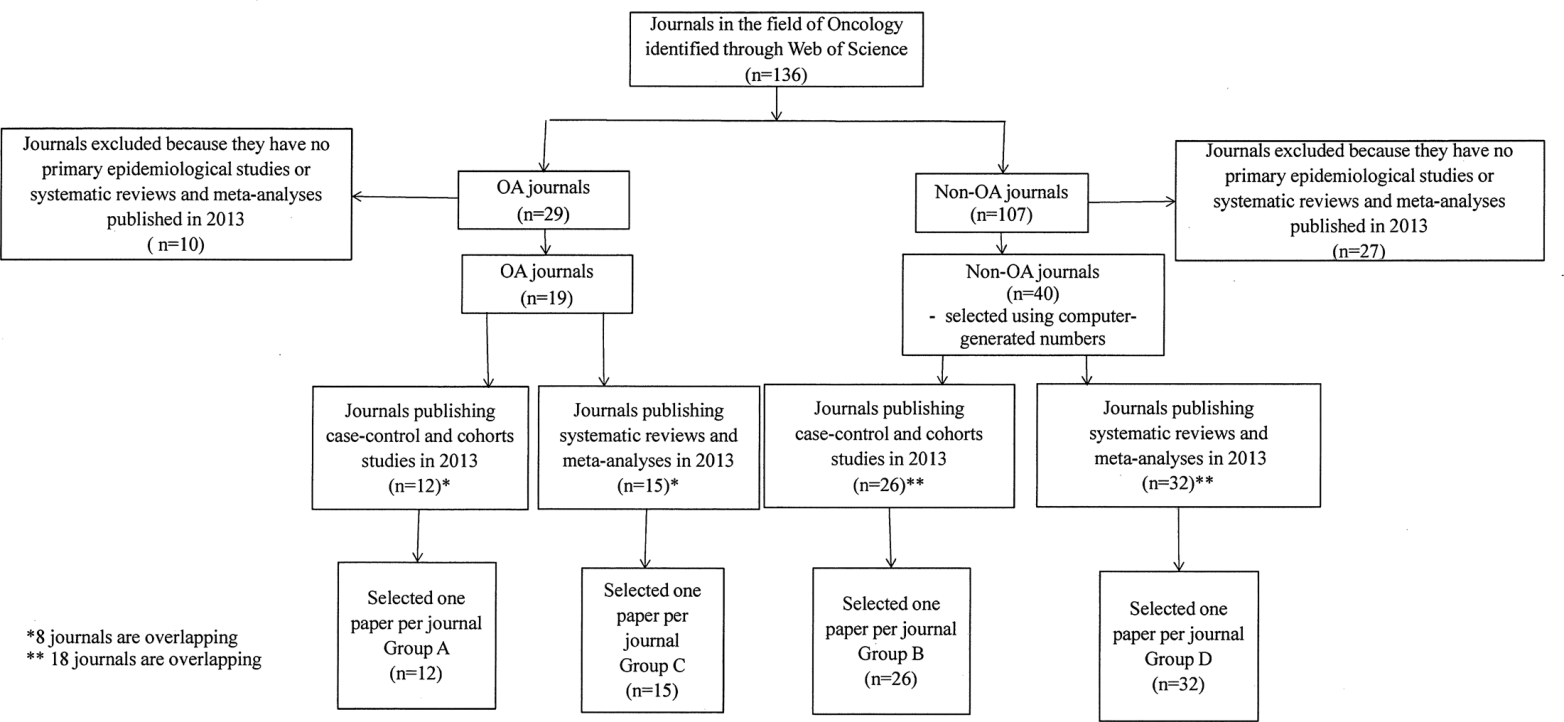
The search strategy and identification process of oncology journals and appraised studies.

One hundred and thirty-six journals publishing English language studies were identified, of which 29 were OA journals and 107 non-OA journals (Fig 1). In order to select eligible articles for the quality appraisal, we selected journals publishing on MEDLINE in 2013 at least one primary epidemiologic study (with a case-control or cohort study design), and at least one systematic review or meta-analysis. Nineteen OA journals met the inclusion criteria of which 12 published case- control and cohort studies, and 15 published systematic reviews and meta-analyses (8 journals were overlapping). Among 80 non-OA eligible journals, we randomly selected (using computer-generated numbers) 40 journals of which 26 published case-control and cohort studies, and 32 published systematic reviews and meta-analyses (18 journals were overlapping).

We then selected the first case-control or cohort study published in 2013 from each of the 12 eligible OA journals (Group A; n = 12), and the first case-control or cohort study published in 2013 from each of the 26 eligible non-OA journals (Group B; n = 26).

We also selected the first systematic review or meta-analysis published in 2013 from each of the 15 eligible OA journals (Group C; n = 15), and the same for each of the 32 non-OA journals (Group D; n = 32).

### Data extraction

From each eligible study we extracted: the name of the journal, the impact factor (IF) of the journal, the name of principal investigator (PI), the country of the PI, the study design, the presence of a supporting source.

### Quality evaluation

The methodological quality of the case-control and cohort studies was evaluated using the Newcastle and Ottawa Scale (NOS) [18], and the quality of the systematic reviews and meta-analyses was assessed by using the Assessment of Multiple Systematic Reviews (AMSTAR) scale [19].

The NOS assigns up to a maximum of 9 points for the least risk of bias in three domains: 1) selection of study groups (four points); 2) comparability of groups (two points); and 3) ascertainment of exposure and outcomes (three points) for case–control and cohort studies, respectively.

The 11 items of AMSTAR scale were rated as “Yes”, “No”, “Cannot answer”, or “Not applicable.” The total score for AMSTAR was calculated by summing one point for each “Yes” and no points for other options, including “No”, “Cannot answer”, and “Not applicable”, resulting in summary scores from 0 to 11.

We assessed the quality of reporting by evaluating the adherence of case-control and cohort studies to the STrengthening the Reporting of OBservational studies in Epidemiology (STROBE) checklist [20], and the adherence of systematic reviews and meta-analyses to the Preferred Reporting Items for Systematic reviews and Meta-Analysis (PRISMA) checklist [21].

Evaluating the adherence to the STROBE and PRISMA checklist was performed by assessing the adherence of each study with the 22-items of the STROBE checklist and with the 27-items of the PRISMA checklist.

Evaluation of each study and the calculations of AMSTAR and NOS total scores were performed in duplicate by three researchers separately (LjE, SM, JS), and in blind with the respect to the status of the journal (OA or non-OA), the name of the authors and the affiliation. In case of disagreement an independent fourth researcher (RP) was consulted.

### Statistical Analysis

In order to evaluate if methodological quality of published studies differs among OA and non-OA oncology journals, we compared Group A versus Group B with the respect to compliance with NOS scale, and Group C versus D with the respect to compliance with AMSTAR scale.

We reported the results as median and interquartile range of NOS score, both overall as well as for each domain and as % of compliance with AMSTAR items, both overall as well as for each item separately.

In order to evaluate if quality of reporting of published studies differs among OA and non-OA oncology journals, we compared Group A versus Group B with the respect to adherence to STROBE Statement, and Group C versus D with the respect to adherence to PRISMA checklist.

We reported the results as % of adherence to the STROBE or PRISMA checklist, both overall as well as for each item separately. For the checklists with more than one recommendation for item, we defined adequate reporting, as an accomplishment to at least 80% of the given recommendations per item. If an item was given by only one recommendation we have defined adequate reporting if the item itself was satisfactory.

Comparison of the percentages between groups was appraised by the Z test for the difference between two proportions or the Fisher test as appropriate, and comparison between continuous variables was evaluated by the nonparametric Wilcoxon rank-sum test.

Additionally, the relation between the quality of the publications and the publication model (OA versus non-OA) was assessed by fitting logistic regression models to the data. As we expected a priori that the quality of publications might differ between journals with largely different values of IF, the regression models were adjusted for IF (included as a continuous variable) to control for potential confounding by this factor.

Concordance between each pair of evaluators was assessed by the Cohen's kappa measure [22].

Statistical analysis was performed using Stata software (StataCorp. 2013. Stata Statistical Software: Release 13. College Station, TX: StataCorp LP).

## Results

Nineteen OA journals and 40 non-OA journals were selected. Among the 19 OA journals, 11 had a for-profit, and 8 a non-profit publishing model.

The median IF of the OA journals that published case-control or cohort studies was 2.2 (IQR 2.1–3.3), compared with 2.8 (IQR 2.1–4.6) of the non-OA journals (p = 0.4, data not shown). The median IF of the OA journals that published systematic reviews or meta-analysis was 3.3 (IQR 2.1–3.3), compared with 2.6 (IQR 1.2–5.2) of the non-OA journals (p = 0.5, data not shown).

Eighty-five studies were deemed eligible for the evaluation. Evaluators’ assessment had a concordance rate greater of 90%, and it did not show a difference between for profit and non-profit OA publishing models (Data not shown).

Overall, 17 (20.0%) out of 85 studies assessed were case-controls, 21 (24.7%) were cohort studies, 18 (21.2%) were systematic reviews and 29 (34.1%) were meta-analyses.

Table 1 reports the characteristics of the 85 studies deemed eligible according to the specific group.

**Table 1:** Characteristics of the 85 studies evaluated published in oncology journals in 2013.

	Group A^§^ (N = 12)	Group B^§§^ (N = 26)	Group C° (N = 15)	Group D°° (N = 32)
**Country provenance**				
Europe	2 (16.7%)	12 (46.2%)	5 (33.3%)	12 (37.5%)
Asia	5 (41.7%)	7 (26.9%)	7 (46.7%)	14 (43.8%)
Australia	0 (0.0%)	1 (3.9%)	1 (6.7%)	1 (3.1%)
America	5 (41.7%)	6 (23.1%)	2 (13.3%)	5 (15.6%)
**Funding source**				
None	4 (33.3%)	4 (15.4%)	8 (53.3%)	22 (68.8%)
Public	8 (66.7%)	11 (42.3%)	6 (40.0%)	7 (21.9%)
Private	0 (0.0%)	2 (7.7%)	0 (0.0%)	3 (9.3%)
Private and Public	0 (0.0%)	9 (34.6%)	1 (6.7%)	0 (0.0%)

§ = case-control and cohort studies published in OA journal.

§§ = case-control and cohort studies published in non-OA journal.

° = systematic review and meta-analysis published in OA journal.

°° = systematic review and meta-analysis published in non-OA journal.

The country distribution was balanced among each of the pairs of compared groups, with a slightly higher prevalence of case-control and cohort studies published from European authors in non-OA journals respect to OA journals.

The vast majority of studies declaring a funding source received from public institutions, without a difference among each pair of groups (Table 1).

### Appraisal of the methodological quality in OA and non-OA journals

The median of NOS score, for case-control and cohort studies appraised in OA and non-OA journals, is reported in Table 2. Results show that there was no difference in the median score in the two groups (Group A: 7 (IQR 7–8) versus Group B: 8 (7–9); p = 0.5), as well as to any particular domain. Even after controlling for IF, the analysis yielded similar result (Overall: p = 0.9; data not shown).

**Table 2:** Assessment of the methodological quality of case-control and cohort studies published in OA and non-OA journals using the Newcastle and Ottawa Scale (NOS).

NOS Domain	Group A^§^ (n = 12)	Group B^§§^ (n = 26)	p-value
Selection	3.5 (3–4)	4 (3–4)	0.3
Comparability	2 (2–2)	2 (1–2)	0.2
Ascertainment	2 (2–3)	2 (2–3)	0.4
**Total**	**7 (7–8)**	**8 (7–9)**	**0.5**

§ = case-control and cohort studies published in OA journal.

§§ = case-control and cohort studies published in non-OA journal.

p-value for the difference in proportions calculated for A vs B

Values are expressed as median and interquartile range of NOS score.

The % of the compliance with AMSTAR scale, for systematic reviews and meta-analyses appraised in OA and non-OA journals is reported in Table 3. Results show that there was a borderline difference between groups concerning the % of the overall compliance, (Group C, 46.0% versus Group D, 55.0%; p = 0.06), however no difference emerged when considering items separately.

**Table 3:** Assessment of the methodological quality of systematic reviews and meta-analyses published in OA and non-OA journals using the Assessment of Multiple Systematic Reviews Scale (AMSTAR).

AMSTAR Item	Group C° (n = 15)	Group D°° (n = 32)	p-value
1	6.7	3.1	0.6
2	53.3	75.0	0.1
3	73.3	78.1	0.7
4	73.3	75.0	0.9
5	0.0	18.8	0.2
6	93.3	93.8	0.9
7	33.3	56.3	0.2
8	26.7	43.8	0.3
9	66.7	78.1	0.7
10	53.3	62.5	0.5
11	26.7	18.8	0.7
**Total (CI 95%)**	**46.0(38.0–54.0)**	**55.0(50.0–60.0)**	**0.06**

° = systematic review and meta-analysis published in OA journal.

°° = systematic review and meta-analysis published in non-OA journal.

p-value for the difference in proportions calculated for C vs D.

Values are expressed as % of compliance to AMSTAR scale.

After controlling for IF, the p-value of the difference in overall compliance between groups increased to 0.9 (data not shown).

### Appraisal of the quality of reporting in OA and non-OA journals

We did not observe significant differences between groups concerning overall adherence to the STROBE checklist (Group A, 75% versus Group B, 80%; p = 0.1) (Table 4), even after controlling for IF (p = 0.3; data not shown). Again, results confirmed that there was no difference in any particular STROBE item (Table 4).

**Table 4:** Proportion of adequate reporting according to the STROBE checklist of the case-control and cohort studies published in OA and non-OA journals using the STrengthening the Reporting of OBservational studies in Epidemiology (STROBE).

STROBE Item	Group A^§^ (n = 12)	Group B^§§^ (n = 26)	p-value
1	100	100	1.0
2	100	100	1.0
3	100	100	1.0
4	91.7	92.3	0.9
5	100	96.2	0.7
6	100	100	1.0
7	66.7	92.3	0.06
8	83.3	96.2	0.2
9	50.0	80.8	0.06
10	25.0	19.2	0.7
11	75.0	88.5	0.3
12	8.3	11.5	0.7
13	16.7	7.7	0.6
14	33.3	34.6	0.9
15	100.0	100.0	1.0
16	100.0	100.0	1.0
17	50.0	73.1	0.2
18	100.0	96.2	0.9
19	75.0	92.3	0.3
20	100.0	100.0	1.0
21	100.0	100.0	1.0
22	83.3	88.5	0.6
**Total (CI 95%)**	**75.0(70.0–80.0)**	**80.0(77.0–83.0)**	**0.1**

§ = case-control and cohort studies published in OA journal.

§§ = case-control and cohort studies published in non-OA journal.

p-value for the difference in proportions calculated for A vs B.

Values are expressed as % of adherence to the STROBE checklist.

Results show that there was no difference between groups concerning overall adherence to the PRISMA checklist (Group C, 72.0% versus Group D, 76.0%; p = 0.1) (Table 5). After controlling for IF, the difference in overall adherence between groups remained non-significant and the p-value increased to 0.4 (data not shown).

**Table 5:** Proportion of adequate reporting according to the Preferred Reporting Items for Systematic reviews and Meta-Analysis (PRISMA) checklist of the systematic reviews and meta-analyses published in OA and non-OA journals.

PRISMA Item	Group C° (n = 15)	Group D°° (n = 32)	p-value
1	93.3	96.9	0.5
2	100.0	90.6	0.5
3	100.0	96.9	0.7
4	73.3	93.8	0.07
5	6.7	3.1	0.5
6	86.7	100.0	0.1
7	100.0	100.0	1.0
8	100.0	93.8	0.5
9	93.3	90.6	0.7
10	66.7	81.3	0.3
11	73.3	78.1	0.7
12	20.0	56.3	0.03 *
13	86.7	78.1	0.6
14	66.7	62.5	0.8
15	53.3	56.3	0.8
16	46.7	43.8	0.9
17	73.3	93.8	0.07
18	100.0	100.0	1.0
19	20.0	50.0	0.06
20	86.7	96.9	0.2
21	60.0	71.8	0.3
22	46.7	43.8	0.8
23	46.7	50.0	0.8
24	100.0	100.0	1.0
25	80.0	71.9	0.7
26	100.0	100.0	1.0
27	60.0	53.1	0.7
**Total (CI 95%)**	**72.0(68.0–76.0)**	**76.0(73.0–79.0)**	**0.1**

° = systematic review and meta-analysis published in OA journal.

°° = systematic review and meta-analysis published in non-OA journal.

p-value for the difference in proportions calculated for C vs D.

Values are expressed as % of adherence to the PRISMA checklist.

* P-value < 0.05 after adjusting for IF

Despite the non-significant result of the overall analysis, we observed a significantly higher rate of adherence to item no. 12 (‘‘Risk of bias in individual studies” under Methods section) of PRISMA checklist for non-OA journals (Group C 20.0% versus Group D 56.3%; p = 0.03) (Table 5). The significant result is confirmed after controlling for IF (p = 0.03; data not shown), although the p-value would not remain significant after adjusting for the number of tests performed.

## Discussion

Our findings indicate that the methodological quality of studies published in OA and non-OA journals, as well as the quality of reporting, are comparable. Across the eligible studies in the field of oncology, we did not observe significant differences among case-control or cohort studies published in OA and non-OA journals, either concerning the compliance with NOS scale and the adherence to STROBE checklist. Additionally, we did not find differences among systematic reviews and meta-analyses, either concerning the compliance with AMSTAR scale and the adherence to PRISMA checklist. The non-significant relation between quality of the publications and publication model (OA versus non-OA) was confirmed after controlling for IF, as we expected a priori that the quality could differ between journals with largely different values of IF.

For almost 15 years, the value and viability of OA journals have been prominent topics of debate in the scientific publishing communities, as the OA publishing has the potential to accelerate recognition and dissemination of research findings, but its actual value is controversial [3–10].

The SOAP (Study of Open Access Publishing) project (http://project-soap.eu/) was financed in 2009 by the European Commission and performed a large-scale survey on the attitudes and experiences of researchers with OA publishing. Around 50,000 answers were collected across disciplines and around the world, showing an overwhelming support for the idea of OA, while highlighting funding and quality as the main barriers to publishing in OA journals. Although 89% percent of respondents answered that OA is beneficial to their field and 53% of respondents reported that they published at least one OA article in their life, around 30% perceived a low quality of the studies currently published in OA journals. Some previous studies attempted to determine the overall quality of OA journals publishing compared to non-OA journals using the citation statistics as main parameter [11–17]. In the scientometrics field, the assumption that citations reflect impact and quality of the studies is built on the idea that novel investigations are based on previously accomplished knowledge, thus on the selection and citation of researches of high quality. Results of these studies showed that OA publishing may reach more readers (as measured by articles downloads) than subscription access publishing papers, but the average citation rates, after controlling for the number of articles, discipline, age of the journal and the location of the publisher are comparable between OA and subscription journals [12,16,17].

However, citations are an indicator of the dissemination of a paper in the scientific community and provide a quantitative system to measure the utilization and contribution of published articles, but they are not strictly linked to the quality of the studies [23].

To our knowledge, this is the first study comparing the methodological quality of studies published in OA and non-OA journals using NOS and AMSTAR scales that have been widely used to judge the overall quality of conduction of observational epidemiologic studies, and systematic reviews and meta-analysis, respectively. Results show that the quality of the studies is identical. Further, we used STROBE and PRISMA checklists to compare the quality of reporting, and again results show no differences among studies published in OA and non-OA journals.

Our study has some limitations. First of all, the topic of oncology was chosen a priori and the analyses were based on the available medical journals in this field. Hence, considering that number of eligible journals of this topic are limited and that the distribution of OA and non-OA journals differs by scientific field, our evaluation is not fully representative of all OA and non-OA journals. Secondly, bearing in mind that the number of OA oncological journals is lower than non-OA journals, the number of studies included in the quality appraisal was limited, and the small sample sizes could limit the power to detect differences between the groups. Lastly, we selected one study per journal, which might have affected the accuracy of our estimates.

## Conclusions

In conclusion, we report that studies published in OA journals in the field of oncology approach the same methodological quality and quality of reporting as studies published in non-OA journals. Additional studies including a larger set of papers and different fields of publication could provide new insights into the quality assessment of studies published in open access and subscription journals.

## Supporting Information

S1 Dataset Dataset with the scores of the different scales. (XLSX)

## Abbreviations

OA: open access
Non-OA: non open access
APC: article processing charges
IF: impact factor
NOS: Newcastle and Ottawa Scale
AMSTAR: Assessment of Multiple Systematic Reviews scale
STROBE: STrengthening the Reporting of OBservational studies in Epidemiology
PRISMA: Preferred Reporting Items for Systematic reviews and Meta-Analysis
